# Comparative Proteomic Analysis of Proteins Involved in Bioenergetics Pathways Associated with Human Sperm Motility

**DOI:** 10.3390/ijms20123000

**Published:** 2019-06-19

**Authors:** Natalina Moscatelli, Paola Lunetti, Clarissa Braccia, Andrea Armirotti, Ferruccio Pisanello, Massimo De Vittorio, Vincenzo Zara, Alessandra Ferramosca

**Affiliations:** 1 Center for Biomolecular Nanotechnologies @UNILE, Istituto Italiano di Tecnologia, Via Barsanti, I-73010 Arnesano (LE), Italy; natalina.moscatelli@unisalento.it (N.M.); ferruccio.pisanello@iit.it (F.P.); massimo.devittorio@iit.it (M.D.V.); 2 Dipartimento di Scienze e Tecnologie Biologiche ed Ambientali, Università del Salento, Via Provinciale Lecce-Monteroni, I-73100 Lecce, Italy; paola.lunetti@unisalento.it (P.L.); vincenzo.zara@unisalento.it (V.Z.); 3 Drug Discovery and Development, Istituto Italiano di Tecnologia, Via Morego, 30-16163 Genova, Italy; clarissa.braccia@iit.it; 4 Analytical Chemistry Lab, Istituto Italiano di Tecnologia, Via Morego, 30-16163 Genova, Italy; andrea.armirotti@iit.it; 5 Dipartimento di Ingegneria Dell’Innovazione, Università del Salento, Via Provinciale Lecce-Monteroni, I-73100 Lecce, Italy

**Keywords:** male infertility, human sperm motility, proteomics, bioenergetics

## Abstract

Sperm motility is the most important parameter involved in the fertilization process and it is strictly required for reproductive success. Although sperm movements are essential for the physiologic fertilization process, the data, deriving from studies focused on the research of altered cell pathways involved in asthenozoospermia, offer only limited information about the molecular mechanism underlying sperm motility. The aim of this study was to identify proteins involved in human sperm motility deficiency by using label-free mass-spectrometry liquid chromatography (LC−MS/MS). For this purpose, we selected sperm samples with three different classes of progressive motility: low, medium (asthenozoospermic samples) and high (normozoospermic samples). We found that several differential expressed proteins in asthenozoospermic samples were related to energetic metabolism, suggesting an interesting link between bioenergetics pathways and the regulation of sperm motility, necessary for the flagellum movement. Therefore, our results provide strong evidence that mass spectrometry-based proteomics represents an integrated approach to detect novel biochemical markers of sperm motility and quality with diagnostic relevance for male infertility and unravel the molecular etiology of idiopathic cases.

## 1. Introduction

Infertility is a multifactorial pathology and more than the 50% of the cases are attributed to the male partner. Male-factor infertility cases are generally idiopathic, and the reasons for this condition are not yet fully understood.

Many studies have suggested the evaluation of different sperm parameters, such as number, morphology, vitality and motility of sperm cells, according to the World Health Organization criteria [1,2,3] but, sometimes, the parameters are inadequate and insufficient to fully understand the reasons for infertility.

In particular, sperm motility is the most important parameter involved in the fertilization process and it is strictly required for the reproductive success. In a sperm sample, three different categories of movement can be discriminated: progressive motility, spermatozoa moving linearly in the field of view, non-progressive motility, with the absence of progression, and immotility [1].

Asthenozoospermia is one of the most common causes of male infertility and subjects affected by this condition usually present total sperm motility under 50% and progressive motility under 32% [4]. Although sperm movements are essential for the physiologic fertilization process, the data, deriving from studies focused on the research of altered cell pathways involved in asthenozospermia offer only limited information about molecular mechanism underlying sperm motility [5,6].

Sperm movement requires energy and this expenditure is supported by two metabolic pathways, glycolysis and oxidative phosphorylation (OXPHOS), that are involved in ATP synthesis, needed for the flagellar beating. Moreover, these pathways are closely linked, with glycolysis feeding the Krebs tricarboxylic acid cycle (TCA), thus sustaining mitochondrial energy production. In particular, during glycolysis, 2 molecules of ATP are produced, while most of ATP molecules (about 32) is produced during cellular respiration. While the number of ATP molecules formed in glycolysis and the TCA cycle is unequivocally known, because it is determined by the stoichiometry of chemical reactions, the number of ATP molecules produced by OXPHOS is less certain, because proton pumping, ATP synthesis and metabolite transport processes can be regulated. Furthermore, since spermatozoa are versatile cells, they could modulate their metabolism according to nutrients availability in female genital tracts [7,8,9].

For several years, different studies have debated the main metabolic way that provides energy to support the motility of sperm cells. A general concept that emerged from all these studies is that spermatozoa exhibit a great versatility in their metabolism: they are indeed able to use different substrates and to activate different energy-producing pathways in dependence of the different environments in which they operate.

The aim of the present study was to identify differences in the expression of proteins involved in the bioenergetic metabolism in human spermatozoa from healthy and asthenozoospermic individuals. To do this, we coupled label-free mass-spectrometry liquid chromatography (LC-MS/MS)-based proteomics and bioinformatics to perform untargeted proteomic analysis of sperm lysates. Proteomic approaches have been already used to identify proteins differently expressed in the comparison between sperm cells produced by fertile and infertile subjects [5,10], but these works did not provide information about metabolic processes related to bioenergetics.

These integrated approaches may drive the identification of novel biochemical markers of sperm motility and quality with diagnostic relevance for male infertility and unravel the molecular etiology of idiopathic cases.

## 2. Results

In cases of abnormal sperm function or molecular defects, it is important to study other sperm characteristics and direct research toward the identification of new potential biomolecular markers. In this context, determining what distinguishes motile from immotile sperm at the proteome level is crucial to appreciate which proteins and cellular pathways play a role in male gamete motility regulation.

### 2.1. Differential Expression of Proteins in Asthenozoospermic and Normozoospermic Sample

A quantitative proteomic comparison between sperm samples with different motility was performed to determine the molecular basis of human sperm motility deficiency. Human sperm samples were selected considering only progressive motility, excluding cases of andrological pathologies. Nine samples pools were constituted by the combination of four samples with similar seminal characteristics.

Human sperm sample pools belonged to three different classes of progressive motility: (i) 44.26 ± 0.17% (normozoospermic samples, *n* = 3), (ii) 24.81 ± 0.30% (asthenozoospermic samples, *n* = 3) and (iii) 12.14 ± 0.17% (severe asthenozoospermic samples, *n* = 3). Pools were treated as described in the “Materials and Methods” section and a high-throughput quantitative proteomics approach was used to identify differences in protein expression in sperms with low (~12%), medium (~24%) and high progressive motility (~44%).

**Figure 1:**
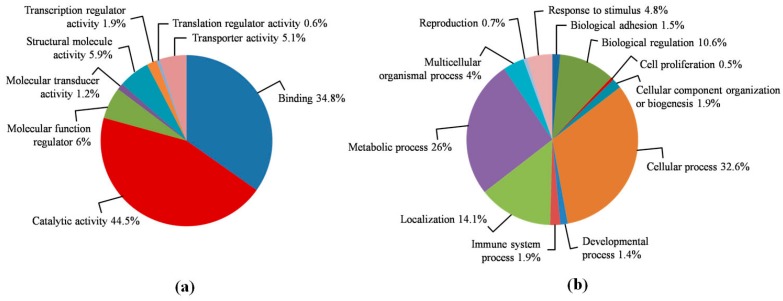
Pie chart of the broad biological functions associated with proteins identified by label-free mass-spectrometry liquid chromatography (LC–MS/MS) in the pooled sperm samples. Molecular functions (**a**) and biological processes (**b**) of the 1962 identified proteins were analyzed by the PANTHER database.

A total of 1962 proteins were identified by LC−MS/MS in the pooled sperm samples. All the identified proteins were classified by biological process and molecular functions by using the PANTHER database. As shown in Figure 1, proteins were grouped into several classes according to their major functions. The major protein classes were enzymes (44.5%) and the binding proteins (34.8%), followed by molecular function regulators (6%), structural molecules (5.9%) and transporters (5.1%) (Figure 1, panel a). These molecules were mainly involved in the following biological processes: cellular processes (cell–cell signaling, cell cycle, growth and proliferation, cell component movement, and cytokinesis; 32.6%), metabolism (26%) and localization (general transport proteins and specific protein and RNA localization processes; 14.3%) (Figure 1, panel b). A subsequent statistical validation comparing low- versus high-motility groups and medium- versus high-motility groups respectively were performed to outline proteins differently expressed between each condition.

**Figure 2:**
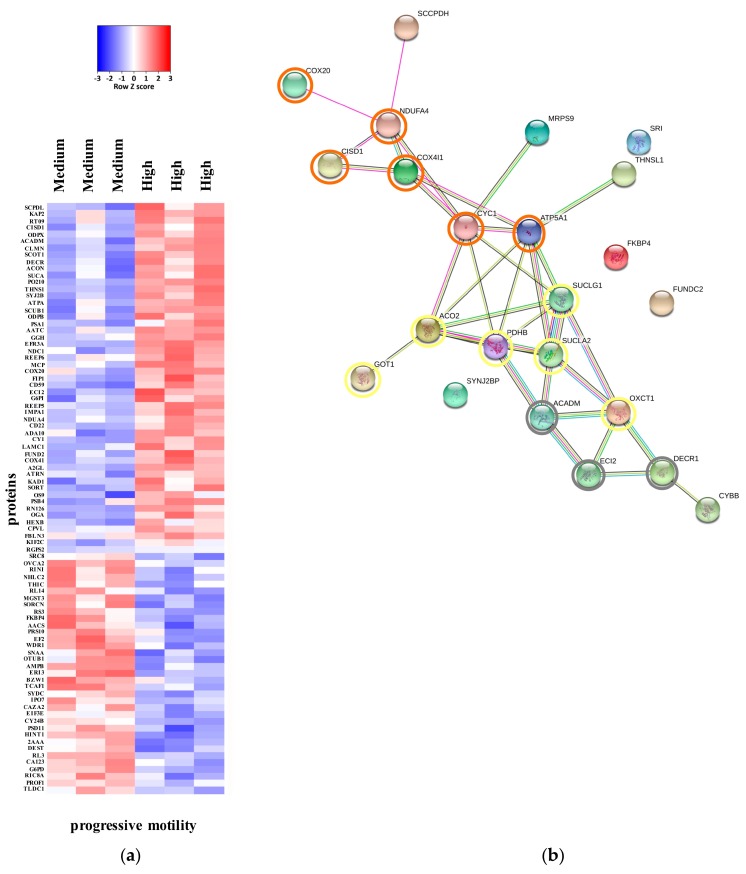
(**a**) Heat map of the expression of proteins which showed *p* < 0.05 in an unpaired, two tails t-test medium group (asthenozoospermic samples with ~24% of progressive motility) vs. high group (normozoosperic samples with ~44% of progressive motility). (**b**) Interactions among the three proteins involved in the polyunsaturated fatty acids catabolism (grey circles), Krebs tricarboxylic acid cycle (TCA) process (yellow circles) and oxidative phosphorylation (OXPHOS) process (orange circles) down-regulated in the human samples with medium progressive motility in comparison to normozoospermic samples. Interactions maps were prepared using the STRING database program. Thicker lines represent stronger associations, and thinner lines represent medium associations.

**Figure 3:**
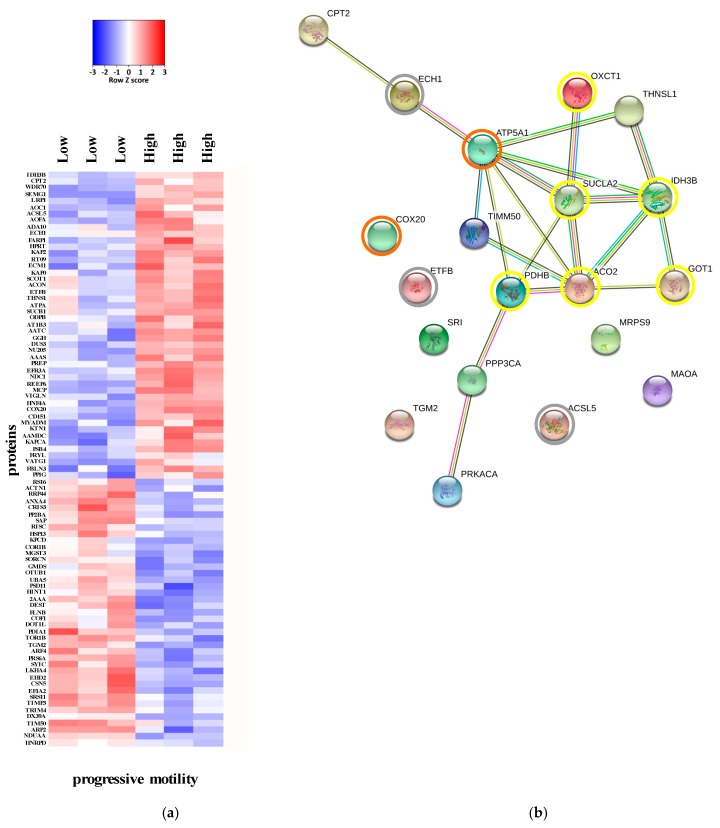
(**a**) Heat map of the expression of proteins which showed *p* < 0.05 in an unpaired, two tails t-test low group (asthenozoospermic samples with ~12% of progressive motility) vs. high group (normozoosperic samples with ~44% of progressive motility). (**b**) Interactions among the four proteins involved in the fatty acids catabolism process (grey circles), TCA process (yellow circles) and OXPHOS process (orange circles) down-regulated in the human samples with low progressive motility in comparison to normozoosperic samples. Interactions maps were prepared using the STRING database. Thicker lines represent stronger associations, and thinner lines represent medium associations.

The comparison of the proteomes of asthenozoospermic and normozoospermic samples resulted in the identification of 86 differentially expressed proteins. 88 proteins were altered when comparing the severe asthenozoospermic and normozoospermic groups (*p* < 0.05). The whole list of differentially expressed proteins is reported in Supplementary Tables (Table S1: Medium vs. High; Table S2: Low vs High). The heat maps in Figure 2 and Figure 3 display differentially expressed proteins that passed *t*- test for statistically significant up- or down-regulation.

### 2.2. Over-Represented Pathway in Asthenozoospermia

Bioinformatics analysis performed by David Bioinformatics Database revealed a considerable number of over-represented pathways, especially involved in energy metabolism. Therefore, proteins involved in fatty acids catabolism, TCA cycle, OXPHOS and electron transport chain (ETC), were found to be down-regulated in asthenozoospermia conditions. The full list of proteins differentially expressed in asthenozoospermic conditions is reported in Table 1 and Table 2. The ratios of protein levels in asthenozoospermics compared to normozoospermics, Medium/High (Table 1) Low/High (Table 2) ranged from 0.18 to 4.81.

Concerning cellular localization, the most represented groups of proteins in both analysis were those localized into the mitochondrion, while as for main cellular functions, a great proportion of the differential proteins have roles in metabolism and energy production.

Metabolic enzymes involved in pyruvate metabolism and TCA cycle were among the most differently regulated proteins. Furthermore, several proteins participating to the mitochondrial ETC complexes, as well as enzymes involved in fatty acids oxidation, are differently expressed.

For these reasons, according to STRING pathways representation almost all significant terms were analyzed considering the three main pathways of energy metabolism: fatty acids catabolism; carbon skeletons catabolism and OXPHOS.

Known and predicted protein-protein interaction networks were represented by STRING interaction maps (Figure 2b and Figure 3b). In these maps, network nodes represent proteins. The associations include direct (physical) interactions, as well as indirect (functional) interactions, as long as both are specific and biologically meaningful.

Thicker lines represent stronger associations, and thinner lines represent medium associations.

#### 2.2.1. Fatty Acids Catabolism

Several proteins involved in lipid metabolism seem to be relevant for spermatogenesis and sperm function [11]. After analyzing the proteome of human sperm samples belonging to three different progressive motility categories, it appears that fatty acid catabolism is the main source of ATP for male gametes. Sperm cells have the enzymatic machinery to perform beta-oxidation of fatty acids of different length and with different unsaturation degrees [11]. From the comparison between medium and high progressive motility, three differentially expressed proteins were identified: ACADM (medium-chain specific acyl-CoA dehydrogenase), ECI2 (enoyl-CoA delta isomerase 2) and DECR1 (2,4-dienoyl-CoA reductase) (Figure 2). In particular, these proteins are involved in catabolism of polyunsaturated fatty acids (PUFA) [12] and their expression is significantly reduced in asthenoozoospermic samples.

Considering the comparison between low and high progressive motility groups, four proteins were identified: CPT2 (carnitine O-palmitoyltransferase 2), ECH1 (delta(3,5)-Delta(2,4)-dienoyl-CoA isomerase), ETFB (electron transfer flavoprotein subunit beta) and ACSL5 (long-chain acyl-CoA synthetase 5) (Figure 3). These proteins are involved in the core of fatty acids catabolism pathway: fatty acid transport in the mitochondrion and beta-oxidation reactions [13].

#### 2.2.2. Carbon Skeletons Catabolism

Little is known about the role of the TCA cycle in the mammalian sperm function, but the close association between this metabolic pathway and the activity of the ETC suggests that an accurate regulation of the TCA cycle in spermatozoa must be ongoing. When comparing the medium progressive motility group with the high one, results showed that six proteins are differentially expressed. Conversely, their expression is significantly reduced in the asthenozoospermic samples. These proteins are: PDHB (pyruvate dehydrogenase E1 component subunit beta), ACO2 (aconitate hydratase), SUCLA2 (succinate-CoA ligase [ADP-forming] subunit beta), SUCLAG1 (succinate-CoA ligase [ADP-forming] subunit beta, hydrogenosomal), OXCT1 (succinyl-CoA:3-ketoacid coenzyme A transferase 1) and GOT1 (aspartate aminotransferase) (Figure 2).

The comparison between low and high progressive motility showed a significant reduction of the expression of the aforementioned proteins, except for SUCLG1 (succinate-CoA ligase [ADP-forming] subunit beta, hydrogenosomal), an enzyme involved in the hydrolysis of succinyl-CoA to the synthesis of ATP (Figure 3) which is slightly downregulated in comparison to normozoospermic samples. In this case, also IDH3B (Isocitrate dehydrogenase [NAD] subunit beta), a regulatory enzyme of TCA, resulted differentially expressed, showing a lower expression degree in severe asthenozospermic samples.

#### 2.2.3. Oxidative Phosphorylation

OXPHOS consists of ETC complexes (complex I-IV) and ATP synthase (complex V) and its function plays a key role in the maintenance of a mitochondrial membrane potential (MMP) across the inner mitochondrial membrane. Proteomic analysis shows low levels of several ETC subunits of complexes I, IV and V in both comparisons.

Moreover, very similar distributions were obtained by selecting proteins according to both cellular localization and function, suggesting that proteins involved in the energetic metabolism, and notably mitochondrial proteins, are typically dysregulated in non-motile sperm.

From the comparison between medium and high progressive motility, differentially expressed proteins, directly and indirectly involved in the OXPHOS process were identified (Figure 2). In particular, CISD1 (CDGSH iron-sulfur domain-containing protein 1), localized to the outer membrane of mitochondria, is thought to play a role in the regulation of oxidation [14], while COX20 (cytochrome *c* oxidase assembly protein COX20) and NDUFA4 (cytochrome *c* oxidase subunit NDUFA4) are involved in the assembly of the complex IV [15,16]. CYC1 (cytochrome *c*_1_), COX4I1, a subunit of the complex IV, and ATP5A1 (ATP synthase subunit alpha), are three proteins directly involved in ATP synthesis [17] and are significantly down-regulated in the medium motility group.

On the other hand, the comparison between low and high progressive motility showed the down-regulation of COX20 and ATP5A1 in the severe asthenozoospermic group (Figure 3).

**Figure 4:**
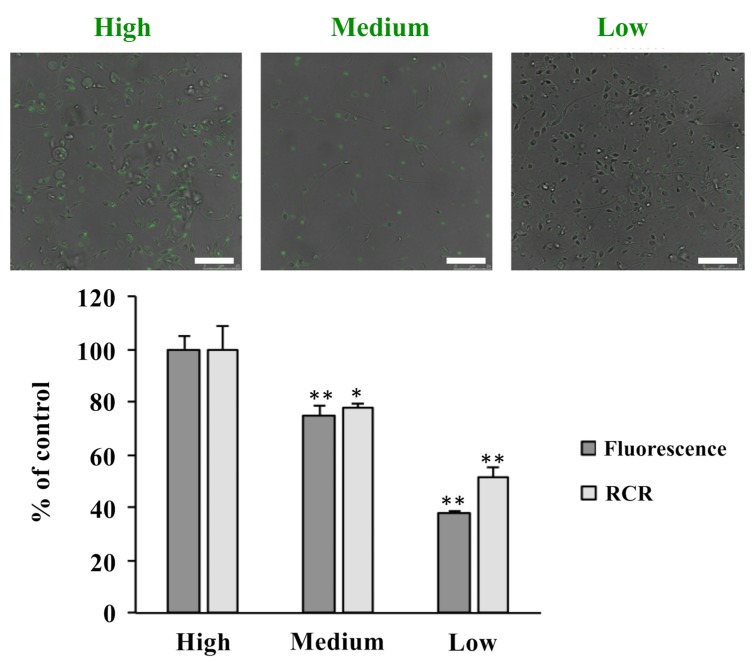
Correlation between sperm progressive motility and fluorescence intensity or respiratory control ratio (RCR) values.

Taken together, these data strongly suggest that the correct functioning of different mitochondrial pathways is critical for the ability of sperm to move. Therefore, mitochondrial function plays a key role in supporting sperm motility and, as a result, a reduced MMP and a reduced respiratory control ratio (RCR) were observed in mitochondria from asthenozoospermic samples in comparison to the control ones (Figure 4).

Samples, treated with MitoTracker Green FM 200 nM, were analyzed by a confocal laser microscope (LEICA TCS SP8 X). Images were acquired with the LasX Software using a 100X oil-immersion objective. The pinhole was set at 1 Airy unit (95 μm). A 490 nm continuous wave diode laser was used for sample excitation with a power of 12%. Fluorescent emission was detected in the spectral window between 500 and 550 nm by a GaAsP photomultiplier tube (PMT); 116.25 × 116.25 μm wide images were acquired at 1024 × 1024 pixels with a pixel size of 113.64 × 113.64 nm, scan speed of 200 Hz. The fluorescence quantification was performed with the open source software ImageJ.

Data are the mean ± standard deviation (SD) of 4 samples of spermatozoa samples with similar characteristics: high, medium and low progressive motility. The values obtained (fluorescence intensity and RCR numbers) from high motility group were set to 100%. Scale bar corresponds to 25 μm.

A *t*-test for statistical comparisons was used.

** *p* < 0.01; *p* (low vs. high) = 0.001393941(fluorescence intensity) and 0.009342592 (RCR value); *p* (medium vs. high) = 0.002197388; * *p* < 0.05; *p* (medium vs. high) = 0.04372016.

## 3. Discussion

Infertility affects 10%–15% of couples, and the male factor is responsible for about half of the infertility cases [18]. Frequently, male infertility is idiopathic and semen analysis alone is not sufficient to distinguish fertile from infertile males. Therefore, it is mandatory to new potential biomarkers of sperm quality and function.

In this context, a proteomic analysis carried out to determine what distinguishes motile from immotile sperm is a crucial approach to evaluate which proteins and cellular pathways play a role in the regulation of male gamete motility. Remarkably, the two lists of differential proteins, deriving from the comparisons between medium and high progressive motility samples and low versus high, share a number of properties. The categorization of proteins, according to both cellular localization and function resulted in very similar distributions, suggesting that mitochondrial proteins involved in energy production are typically deregulated in sperm from asthenozoospermic subjects. Similar alterations in the proteomic composition were found in the two comparisons (medium versus high and low versus high motility), providing an evidence that these protein families/cellular pathways might indeed be involved in sperm motility regulation.

As anticipated, many proteins dysregulated in sperm samples with motility defects play important roles in the energetic metabolism, since ATP is evidently needed to support sperm motility. Indeed, most of the pathways detected by the David Bioinformatics Database were related to energy metabolism.

It was indeed interesting to determine that low-motility sperm have altered levels of so many post-glycolytic enzymes, which clearly suggests that glycolysis may not be the main actor in sperm motility maintenance. It is interesting to note that the glycolytic enzymes, as well as enzymes and proteins involved in the lactate metabolism, do not undergo significant variations in the three classes of motility. These data confirm the key role played by mitochondria in sperm physiology and motility [7,19], since these organelles are not only the location of specific metabolic pathways related to energy production as in other cell types, but they are characterized by peculiar kinetic and regulatory properties [7]. The analysis of proteomic profile related to sperm motility shows that the level of enzymes and proteins involved in the mitochondrial energy pathways decreases when the motility is reduced. This is the first evidence of a bioenergetic adaptation mechanism, in terms of expression of proteins involved in mitochondrial bioenergetics, used by spermatozoa to support cell motility.

In agreement, the levels of mitochondrial complex IV subunits were positively correlated with human sperm motility [20]; moreover, the inhibition of mitochondrial beta-oxidation impairs human sperm motility, confirming the key role of mitochondrial fatty acid catabolism, at least in the absence of exogenous sugars [11].

Considering the comparisons of progressive motility, medium versus high and low versus high, seven proteins resulted in being downregulated in both asthenozoospermic groups. Pyruvate dehydrogenase E1 component subunit beta (PDHB), Aconitate hydratase (ACO2), Succinate-CoA ligase [ADP-forming] subunit beta (SUCLA2), Succinyl-CoA:3-ketoacid coenzyme A transferase (OXCT1) and Aspartate aminotransferase (GOT1) are enzymes involved in the carbon skeletons catabolism, while ATP synthase subunit alpha (ATP5A1) and Cytochrome *c* oxidase assembly protein COX20 (COX20) are subunits of the ETC. In detail, PDHB is a subunit of a nuclear-encoded mitochondrial multienzyme complex responsible for the conversion of pyruvate to acetyl-CoA and CO_2_, which provides the primary link between glycolysis and the TCA cycle. ACO2 is an enzyme involved in the second step of the TCA cycle, that catalyzes the interconversion of citrate to isocitrate. Little is known about the role and mechanism of ACO2 in asthenozoospermia, although immunoblotting experiments demonstrated that the level of ACO2 protein in the asthenozoospermic samples was significantly decreased in comparison to normal fertile men [21]. It is interesting to underline that mitochondrial aconitase activity could be considered a functional indicator of mitochondrial levels of reactive oxygen species (ROS), because the iron-sulfur core of this enzyme is oxidized by superoxide anions, reducing its activity [22]. We can therefore speculate that the observed control of aconitase expression is a mechanism to prevent a condition of oxidative stress. SUCLA2 is a mitochondrial matrix enzyme that catalyzes the conversion of succinyl-CoA to succinate and acetoacetyl-CoA, and the phosphorylation of ADP to ATP, in the TCA cycle. OXCT1 plays a role in energy metabolism and may indicate the existence of a novel metabolic system utilizing ketone bodies as an energy source for sperm motility [23]. GOT1 shuttles NADH into the mitochondrial matrix [7], ATP5A1 is a catalytic subunit of the ATP-synthase and the increased expression of the ATP synthase subunit alpha in the high-motility sperm suggests that this enzyme may represent a new molecular marker of the evaluation of sperm motility [24]. COX20 is a complex IV subunit, not directly involved in the ATP synthesis, but important for the correct assembly of the complex itself [15]. In the two classes of reduced motility we observed a decrease of a subunit of the ATP synthase and of a subunit involved in the assembly of the complex IV, the last complex of the respiratory chain, the target of different flows of reducing equivalents, coming from different metabolic pathways. On the other hand, in the medium group we observed variations in the expression levels of many proteins involved, directly or indirectly, in the OXPHOS process. According to the proteomic profile observed in asthenozoosperic samples, a relationship between mitochondrial respiration efficiency and sperm motility was already proposed in previous studies [25,26] and also in the present study we observed mitochondrial respiratory defects when asthenozospermic samples were subjected to fluorescence or to polarographic assays.

Our results suggest that all the proteins differentially expressed in both groups of asthenozoospermic samples and related to the bioenergetic pathways may be closely linked to the regulation of progressive motility, necessary for the flagellum movement. Therefore, mass spectrometry-based proteomics represents a powerful tool to detect proteins and pathways in view of their potential diagnostic and prognostic use and perhaps to determine novel therapeutic targets.

## 4. Materials and Methods

### 4.1. Human Semen Samples

For this study, sperm cells samples and respective spermiograms were provided by the biological medical center “Tecnomed” in Nardò (Lecce), Italy. The study was conducted in accordance with the Declaration of Helsinki, and the research (New biochemical markers for the nanodiagnostic of male infertility) was approved by the Institutional Review Board of Department of biological and environmental sciences and technologies at the University of Salento (10 November 2015). All experiments were performed in accordance with the relevant guidelines and regulations for research on human subjects. The use of semen was allowed by the donors who signed a written informed consent.

Patients (between 24 and 47 years of age) did not have any conditions interfering with semen analysis, such as urogenital infections, leukocytospermia or systemic diseases; moreover, they did not have a history of smoking or alcohol abuse or drug consumption, and did not receive antioxidant supplements or medication with proven toxicity on fertility.

Automated computer analysis of sperm motility (CASA-SCA: Sperm Class Analyzer) was carried out on all semen samples. The motility of each spermatozoon was graded according to the World Health Organization (WHO) laboratory manual for the examination and processing of human semen [1]. In particular, progressive motility included spermatozoa moving actively, either linearly or in a large circle, regardless of speed.

### 4.2. Sample Preparation: Cell Lysis and Protein Digestion

For these experiments, sperm samples were selected by evaluating only the progressive motility and were pooled considering three different classes of progressive motility: ~44%, ~24% and ~12%. Each pool consisted of four sperm samples with similar characteristics.

After pooling, samples were centrifuged at 10,000× *g* for 5 min at 4 °C, in order to separate cells from the seminal fluids. Supernatants were discarded and sperm pellets were incubated for an hour, at room temperature, with a protein extraction buffer containing UREA 6M, Di-thiothreitol (DTT) 18.6 mM and 2% CHAPS. Then samples were centrifuged again at 12,000× *g* for 5 min at 4 °C and supernatants, with extracted proteins, were stored at –80 °C until use. Protein concentration for each samples pool, was determined by Bradford method [27].

The volume corresponding to 50 µg of proteins from sperm lysates was reduced with 10 µl of 100 mM DTT (56 °C for 30 min), then alkylated with 30 µl of 100 mM iodoacetamide (20 min at room temperature in the dark). The samples were then precipitated in cold acetone (1 mL, −20 °C) overnight then centrifuged at 15,000× *g* for 30 min at 4 °C. The supernatant was discarded, and the pellet was dried under nitrogen stream before trypsin digestion. An overnight digestion was performed at 37 °C in 50 mM NH_4_HCO_3_ in MilliQ water (pH 8.0) with 1 µg of trypsin. The resulting peptides were dried under vacuum and then dissolved in 150 µL of 3% acetonitrile (ACN) + 0.1% formic acid (FA).

### 4.3. Label-Free Mass-Spectrometry Liquid Chromatography (LC–MS/MS) Analysis

The same amount of peptides (1.66 µg) was analyzed by high-resolution LC–MS analysis using a NanoAcquity chromatographic system coupled to a TripleTof 5600+ mass spectrometer equipped with a NanoSpray III ion source. The peptides were loaded and desalted on a trapping column (180 µm × 20 mm Acquity C18) for 4 min at 4.0 µL/min flow rate (1% ACN + 0.1% FA), and then separated on a PicoFrit C18 column (75 μm x 25 cm, from NewObjective Inc., Woburn, MA, USA). The peptides were eluted with a 2-h linear gradient at 300 nL/min (from 3% to 45% of ACN in water + 0.1% FA); the column was washed with 90% ACN and then equilibrated for 18 min to 3% ACN.

The mass spectrometer parameters were set as follow: ion spray voltage at 2500 V, spray gas 1: 10, curtain gas: 30, declustering potential: 80 V and source temperature: 90 °C.

The spectra for protein quantification were acquired in data-independent acquisition (DIA) mode, following the SWATH protocol for label free proteomics [28]. The SWATH variable window width was set from 7 to 50 Da and the precursors ions were selected in the 400–1250 *m*/*z* range with a full range survey scan of 250 ms. 100 consecutive SWATH experiments (each lasting 25 ms) were performed in the 100–1500 *m*/*z* range.

### 4.4. Proteins Quantification

The DIA spectra were searched against the PanHuman ion library [29], using only non-shared peptides. For the quantification, the following criteria were used: minimum peptide confidence 90%, 50 ppm maximum mass tolerance, 60 min maximum RT tolerance, 6 MRM transitions per peptide and modified peptides were not allowed. With these settings, we were able to quantify 1959 from cell lysates.

### 4.5. Data Analysis

For statistical analysis, raw data were imported in MarkerView software and normalized using most likely ratio (MLR) method [30]. To select only the significantly (*p* value < 0.05) dysregulated proteins, three one-to-one comparisons were performed using an un-paired, 2 tails *t*-test.

### 4.6. Pathway Analysis

Biological processes and molecular functions of the identified proteins were analyzed by the PANTHER database (http://pantherdb.org/).

Network and pathway analysis was performed using STRING (https://string-db.org/) [31] and David Bioinformatics Database (https://david.ncifcrf.gov) with the following parameters: medium confidence (score 0.4) for all the interactive sources and with line thickness indicating the strength of interaction. All the interaction sources were selectedas data mining parameters (textmining, experiments, databases, co-expression, neighborhood, gene fusion and co-occurrence). Disconnected nodes in the network were hidden for clarity purposes.

### 4.7. Mitochondrial Function Evaluation

Spermatozoa were collected by centrifugation at 800× *g* for 10 min at room temperature, washed by resuspension in isotonic salt medium, subjected to hypotonic treatment and used for polarographic assays of oxygen consumption [32]. The RCR was calculated by dividing V_3_ (rate of oxygen uptake measured in the presence of respiratory substrates (10 mM pyruvate, 10 mM malate and 0.76 mM ADP) by V_4_ (rate of oxygen uptake measured with respiratory substrates pyruvate and malate alone).

In order to assess MMP, staining of sperm mitochondria with MitoTracker Green FM and laser scanning confocal (LSC) analysis was carried out as described in [33].

## Figures and Tables

**Table 1:** Description of proteins involved in energy metabolism, which showed a significant p.value (*p* < 0.05) in an unpaired, two tails *t*-test medium vs high. Results are expressed as ratios of protein levels of asthenozoospermics to normozoospermics.

UniProt ID	Gene Name	Protein Name	Medium/High
Q8IYQ7	*THNSL1*	Threonine synthase-like 1	0.19
P82933	*MRSP9*	28S ribosomal protein S9	0.26
Q9BWH2	*FUNDC2*	FUN (Function Unkown Now) 14 domain-containing protein 2	0.28
Q5RI15	*COX20*	Cytochrome *c* oxidase assembly protein COX20	0.31
P53587	*SUCLG1*	Succinate-CoA ligase [ADP-forming] subunit beta, hydrogenosomal	0.31
Q8NBX0	*SCCPDH*	Saccharopine dehydrogenase-like oxidoreductase	0.37
Q9P2R7	*SUCLA2*	Succinate-CoA ligase [ADP-forming] subunit beta	0.37
P11310	*ACADM*	Medium-chain specific acyl-CoA dehydrogenase	0.41
Q99798	*ACO2*	Aconitate hydratase	0.42
P55809	*OXCT1*	Succinyl-CoA:3-ketoacid coenzyme A transferase 1	0.43
P08574	*CYC1*	Cytochrome *c*_1_	0.44
Q16698	*DECR1*	2,4-dienoyl-CoA reductase	0.48
O75521	*ECI2*	Enoyl-CoA delta isomerase 2	0.50
P11177	*PDHB*	Pyruvate dehydrogenase E1 component subunit beta	0.55
Q9NZ45	*CISD1*	CDGSH iron-sulfur domain-containing protein 1	0.56
O00483	*NDUFA4*	Cytochrome *c* oxidase subunit NDUFA4	0.56
P17174	*GOT1*	Aspartate aminotransferase	0.59
P25705	*ATP5A1*	ATP synthase subunit alpha	0.60
P57105	*SYNJ2BP*	Synaptojanin-2-binding protein	0.61
P13073	*COX4I1*	Cytochrome *c* oxidase subunit 4 isoform 1	0.66
Q02790	*FKBP4*	Peptidyl-prolyl cis-trans isomerase FKBP4	1.50
P04839	*CYBB*	Cytochrome *b*-245 heavy chain	2.27
P30626	*SRI*	Sorcin	4.81

**Table 2:** Description of proteins involved in energy metabolism, which showed a significant *p*.value (*p* < 0.05) in an unpaired, two tails *t*-test low vs high. Results are expressed as ratios of protein levels of severe asthenozoospermics to normozoospermics.

UniProt ID	Gene Name	Protein Name	Low/High
P82933	*MRPS9*	28S ribosomal protein S9	0.18
Q5RI15	*COX20*	Cytochrome c oxidase assembly protein	0.22
Q9ULC5	*ACSL5*	Long-chain acyl-CoA synthetase 5	0.32
P21397	*MAOA*	Amine oxidase [flavin-containing] A	0.32
Q8IYQ7	*THNSL1*	Threonine synthase-like 1	0.45
P17174	*GOT1*	Aspartate aminotransferase	0.48
P17612	*PRKACA*	cAMP-dependent protein kinase catalytic subunit alpha	0.58
P38117	*ETFB*	Electron transfer flavoprotein subunit beta	0.62
Q99798	*ACO2*	Aconitate hydratase	0.63
Q9P2R7	*SUCLA2*	Succinate-CoA ligase [ADP-forming] subunit beta	0.64
P11177	*PDHB*	Pyruvate dehydrogenase E1 component subunit beta	0.65
P55809	*OXCT1*	Succinyl-CoA:3-ketoacid coenzyme A transferase 1	0.66
P23786	*CPT2*	Carnitine O-palmitoyltransferase 2	0.67
P25705	*ATP5A1*	ATP synthase subunit alpha	0.68
O43837	*IDH3B*	Isocitrate dehydrogenase [NAD] subunit beta	0.68
Q13011	*ECH1*	Delta(3,5)-Delta(2,4)-dienoyl-CoA isomerase	0.85
Q3ZCQ8	*TIMM50*	Mitochondrial import inner membrane translocase subunit TIM50	2.06
Q08209	*PPP3CA*	Serine/threonine-protein phosphatase 2B catalytic subunit alpha isoform	2.58
P21980	*TGM2*	Protein-glutamine gamma-glutamyltransferase 2	3.19
P30626	*SRI*	Sorcin	3.60

## Supplementary Materials

Supplementary materials can be found at https://www.mdpi.com/1422-0067/20/12/3000/s1.
