# Supplementary material for: Comparative Proteomic Analysis of Proteins Involved in Bioenergetics Pathways Associated with Human Sperm Motility
Source: Int J Mol Sci. 2019 Jun 19;20(12):3000. doi: 10.3390/ijms20123000 (PMC6627292; doi:10.3390/ijms20123000)
Supplement: Supplementary file 1 [file ijms-20-03000-s001.pdf]

Supplementary Materials

Table S1. Details of proteins reported in Figure 2 panel A. List of proteins up- and down-regulated in samples with medium progressive motility with respect to high progressive motility group identified by LC-MS/MS analysis. Results are expressed as ratios of protein levels of asthenozoospermics to normozoospermics.

UniProt ID	Gene Name	Protein Name	Medium/High
**P20273**	*CD22*	B-cell receptor CD22	0.14
**Q8IYQ7**	*THNS1*	Threonine synthase-like 1	0.19
**Q99523**	*SORT*	Sortilin	0.22
**P82933**	*RT09*	28S ribosomal protein S9	0.26
**Q14156**	*EFR3A*	Protein EFR3 homolog A	0.27
**Q9BWH2**	*FUND2*	FUN14 domain-containing protein 2	0.28
**Q5RI15**	*COX20*	Cytochrome c oxidase assembly protein COX20	0.31
**P53597**	*SUCA*	Succinate-CoA ligase	0.31
**Q9BTX1**	*NDC1*	Nucleoporin NDC1	0.33
**P15529**	*MCP*	Membrane cofactor protein (TLX)	0.35
**Q8TEM1**	*PO210*	Nuclear pore membrane glycoprotein 210	0.36
**Q8NBX0**	*SCPDL*	Saccharopine dehydrogenase-like oxidoreductase	0.37
**Q9P2R7**	*SUCB1*	Succinate-CoA ligase [ADP-forming] subunit beta	0.37
**Q99661**	*KIF2C*	Kinesin-like protein KIF2C	0.38
**P02750**	*A2GL*	Leucine-rich alpha-2-glycoprotein	0.39
**Q6UN15**	*FIP1*	Pre-mRNA 3'-end-processing factor FIP1	0.40
**P11310**	*ACADM*	Medium-chain specific acyl-CoA dehydrogenase	0.41
**Q99798**	*ACON*	Aconitate hydratase	0.42
**P55809**	*SCOT1*	Succinyl-CoA:3-ketoacid coenzyme A transferase 1	0.43
**P08574**	*CY1*	Cytochrome c1	0.44
**O14672**	*ADA10*	Disintegrin and metalloproteinase domain-containing protein 10	0.46
**Q13438**	*OS9*	Protein OS-9	0.46
**Q16698**	*DECR*	2,4-dienoyl-CoA reductase	0.48
**Q92820**	*GGH*	Gamma-glutamyl hydrolase	0.48
**O75521**	*ECI2*	Enoyl-CoA delta isomerase 2	0.50
**Q9BV68**	*RN126*	E3 ubiquitin-protein ligase RNF126	0.50
**O75882**	*ATRN*	Attractin	0.51
**P11310**	*CLMN*	Calmin	0.52
**P11047**	*LAMC1*	Laminin subunit gamma-1	0.54
**P00568**	*KAD1*	Adenylate kinase isoenzyme 1	0.54
**P11177**	*ODPB*	Pyruvate dehydrogenase E1 component subunit beta	0.55
**O60502**	*OGA*	Protein O-GlcNAcase	0.55
**Q9NZ45**	*CISD1*	CDGSH iron-sulfur domain-containing protein 1	0.56
**O00483**	*NDUA4*	Cytochrome c oxidase subunit NDUFA4	0.56
**P06744**	*G6PI*	Saccharopine dehydrogenase-like oxidoreductase	0.57
**Q9H3G5**	*CPVL*	Probable serine carboxypeptidase CPVL	0.58
**P29218**	*IMPA1*	Inositol monophosphatase 1	0.58
**P17174**	*AATC*	Aspartate aminotransferase	0.59
**P28070**	*PSB4*	Proteasome subunit beta type-4	0.60
**P13987**	*CD59*	CD59 glycoprotein	0.60
**P25705**	*ATPA*	ATP synthase subunit alpha	0.60
**P57105**	*SYJ2B*	Synaptojanin-2-binding protein	0.61
**Q00765**	*REEP5*	Receptor expression-enhancing protein 5	0.61
**P07686**	*HEXB*	Beta-hexosaminidase subunit beta	0.63
**P0C7H8**	*KAP2*	Keratin-associated protein 2-3	0.63
**P0CO20**	*PSA1*	Mannose-1-phosphate guanyltransferase	0.64
**P13073**	*COX41*	Cytochrome c oxidase subunit 4 isoform 1	0.67
**Q96HR9**	*REEP6*	Receptor expression-enhancing protein 6	0.68
**Q12805**	*FBLN3*	EGF-containing fibulin-like extracellular matrix protein 1	0.69
**O00330**	*ODPX*	Pyruvate dehydrogenase protein X component	0.71
**Q86X27**	*RGPS2*	Ras-specific guanine nucleotide-releasing factor RalGPS2	0.74
**P54920**	*SNAA*	Alpha-soluble NSF attachment protein	1.23

**P07737**	*PROF1*	Profilin-1	1.30
**Q8WU68**	*RS3*	Splicing factor U2AF 26 kDa subunit	1.32
**O00231**	*PSD11*	26S proteasome non-ATPase regulatory subunit 11	1.34
**Q9H4A4**	*AMPB*	Aminopeptidase B	1.35
**P13639**	*EF2*	Elongation factor 2	1.36
**P62333**	*PRS10*	26S proteasome regulatory subunit 10B	1.37
**P14868**	*SYDC*	Aspartate-tRNA ligase	1.47
**Q02790**	*FKBP4*	Peptidyl-prolyl cis-trans isomerase FKBP4	1.50
**Q14204**	*2AAA*	Cytoplasmic dynein 1 heavy chain 1	1.55
**O75083**	*WDR1*	WD repeat-containing protein 1	1.55
**P15144**	*RINI*	Aminopeptidase N	1.56
**I3B4L0**	*THIC*	Multifunctional fusion protein	1.57
**P60228**	*EIF3E*	Eukaryotic translation initiation factor 3 subunit E	1.57
**Q8WZ82**	*OVCA2*	Esterase OVCA2	1.61
**P11413**	*G6PD*	Glucose-6-phosphate isomerase	1.69
**O43414**	*ERI3*	ERI1 exoribonuclease 3	1.75
**P49773**	*HINT1*	Histidine triad nucleotide-binding protein 1	1.76
**P60981**	*DEST*	Destrin	1.77
**Q96FW1**	*OTUB1*	Ubiquitin thioesterase OTUB1	1.78
**Q14247**	*SRC8*	Src substrate cortactin	1.90
**O95373**	*IPO7*	Importin-7	1.94
**Q7L1Q6**	*BZW1*	Basic leucine zipper and W2 domain-containing protein 1	1.96
**P50914**	*RL14*	60S ribosomal protein L14	1.97
**P47755**	*CAZA2*	F-actin-capping protein subunit alpha-2	2.06
**Q96RI0**	*RL3*	Proteinase-activated receptor 4	2.12
**P04839**	*CY24B*	Cytochrome b-245 heavy chain	2.27
**Q86V21**	*AACS*	Acetoacetyl-CoA synthetase	2.28
**Q0Q8R6**	*CA123*	Capsid scaffolding protein	2.38
**Q9NPQ8**	*RIC8A*	Synembryn-A	2.44
**O14880**	*MGST3*	Microsomal glutathione S-transferase 3	2.91
**Q7L1Q6**	*TCAF1*	TRPM8 channel-associated factor 1	3.01
**Q6P9B6**	*TLDC1*	TLD domain-containing protein 1	3.14
**Q8NBF2**	*NHLC2*	NHL repeat-containing protein 2	3.14
**P30626**	*SORCN*	Sorcin	4.81

Table S2. Details of proteins reported in Figure 3 panel A. List of proteins up- and down-regulated in samples with low progressive motility with respect to high progressive motility group identified by LC-MS/MS analysis. Results are expressed as ratios of protein levels of severe asthenozoospermics to normozoospermics.

**UniProt ID**	**Gene Name**	**Protein Name**	**Low/High**
**P04279**	*SEMG1*	Semenogelin-1	0.00
**P19801**	*AOC1*	Amiloride-sensitive amine oxidase	0.10
**Q9NW82**	*WDR70*	WD repeat-containing protein 70	0.14
**Q9H7C9**	*AAMDC*	Mth938 domain-containing protein	0.16
**P15529**	*MCP*	Membrane cofactor protein	0.17
**P82933**	*RT09*	28S ribosomal protein S9	0.18
**Q5RI15**	*COX20*	Cytochrome c oxidase assembly protein COX20	0.22
**P48509**	*CD151*	CD151 antigen	0.24
**Q12805**	*FBLN3*	EGF-containing fibulin-like extracellular matrix protein 1	0.27
**Q96S97**	*MYADM*	Myeloid-associated differentiation marker	0.29
**P21397**	*AOFA*	Amine oxidase [flavin-containing] A	0.32
**Q9ULC5**	*ACSL5*	Long-chain-fatty-acid-CoA ligase 5	0.32
**Q07954**	*LRP1*	Prolow-density lipoprotein receptor-related protein 1	0.33

**Q92820**	*GGH*	Gamma-glutamyl hydrolase	0.33
**P41235**	*HNF4A*	Hepatocyte nuclear factor 4-alpha	0.35
**Q9BTX1**	*NDC1*	Nucleoporin NDC1	0.38
**Q92621**	*NU205*	Nuclear pore complex protein	0.38
**Q14156**	*EFR3A*	Protein EFR3 homolog A	0.39
**Q9Y4F1**	*FARP1*	FERM, ARHGEF and pleckstrin domain-containing protein 1	0.42
**P36873**	*PP1G*	Protein phosphatase 1 regulatory subunit 3A	0.42
**O94915**	*FRYL*	Protein furry homolog-like	0.44
**Q9NRG9**	*AAAS*	Aladin	0.44
**Q8IYQ7**	*THNS1*	Threonine synthase-like 1	0.45
**Q96HR9**	*REEP6*	Receptor expression-enhancing protein 6	0.48
**Q86UP2**	*KTN1*	Kinectin	0.48
**P17174**	*AATC*	Aspartate aminotransferase	0.48
**P54709**	*AT1B3*	Sodium/potassium-transporting ATPase subunit beta-3	0.49
**Q5JRX3**	*PREP*	Prolyl endopeptidase	0.50
**Q00341**	*VIGLN*	Vigilin	0.51
**P51452**	*DUS3*	tRNA-dihydrouridine(47) synthase	0.53
**Q16610**	*ECM1*	Extracellular matrix protein 1	0.54
**P13861**	*KAP2*	Keratin-associated protein 2-3	0.55
**O75348**	*VATG1*	V-type proton ATPase subunit G 1	0.55
**P17612**	*KAPCA*	cAMP-dependent protein kinase catalytic subunit alpha	0.58
**P38117**	*ETFB*	Electron transfer flavoprotein subunit beta	0.62
**Q99798**	*ACON*	Aconitate hydratase	0.63
**P28070**	*PSB4*	Proteasome subunit beta type-4	0.63
**P10644**	*KAP0*	cAMP-dependent protein kinase type I-alpha regulatory subunit	0.63
**O14672**	*ADA10*	Disintegrin and metalloproteinase domain-containing protein 10	0.63
**Q9P2R7**	*SUCB1*	Succinate-CoA ligase [ADP-forming] subunit beta	0.64
**P11177**	*ODPB*	Pyruvate dehydrogenase E1 component subunit beta	0.65
**P55809**	*SCOT1*	Succinyl-CoA:3-ketoacid coenzyme A transferase 1	0.66
**P23786**	*CPT2*	Carnitine O-palmitoyltransferase 2	0.67
**O43837**	*IDH3B*	Isocitrate dehydrogenase [NAD] subunit beta	0.68
**P25705**	*ATPA*	ATP synthase subunit alpha	0.68
**P00492**	*HPRT*	Hypoxanthine-guanine phosphoribosyltransferase	0.70
**Q13011**	*ECH1*	Delta(3,5)-Delta(2,4)-dienoyl-CoA isomerase	0.85
**P61160**	*ARP2*	Actin-related protein 2	1.25
**P12814**	*ACTN1*	Alpha-actinin-1	1.25
**O00231**	*PSD11*	26S proteasome non-ATPase regulatory subunit 11	1.31
**P07602**	*SAP*	SH2 domain-containing protein 1A	1.34
**Q9BR76**	*COR1B*	Coronin-1B	1.35
**P62249**	*RS16*	40S ribosomal protein S16	1.40
**P48723**	*HSP13*	Heat shock 70 kDa protein 13	1.42
**P18085**	*ARF4*	ADP-ribosylation factor 4	1.45
**P17980**	*PRS6A*	26S proteasome regulatory subunit 6A	1.50

**P23528**	*COF1*	Cofilin-1	1.50
**Q9HB40**	*RISC*	Protein argonaute-2	1.52
**Q14103**	*HNRPD*	Heterogeneous nuclear ribonucleoprotein D0	1.55
**P49773**	*HINT1*	Histidine triad nucleotide-binding protein 1	1.58
**Q96FW1**	*OTUB1*	Ubiquitin thioesterase OTUB1	1.60
**O60547**	*GMDS*	GDP-mannose 4,6 dehydratase	1.62
**Q9C037**	*TRIM4*	E3 ubiquitin-protein ligase TRIM4	1.63
**P30153**	*2AAA*	Cytoplasmic dynein 1 heavy chain 1	1.66
**O75369**	*FLNB*	Protein disulfide-isomerase	1.72
**P07237**	*PDIA1*	Protein disulfide-isomerase	1.79
**Q05655**	*KPCD*	Protein kinase C delta type	1.79
**P09525**	*ANXA4*	Annexin A4	1.83
**Q9Y2L1**	*RRP44*	Exosome complex exonuclease RRP44	1.85
**P60981**	*DEST*	Destrin	1.91
**P41252**	*SYIC*	Isoleucine-tRNA ligase	1.93
**Q05519**	*SRS11*	Serine/arginine-rich splicing factor 11	1.97
**P35625**	*TIMP3*	Metalloproteinase inhibitor 3	2.06
**Q3ZCQ8**	*TIM50*	Mitochondrial import inner membrane translocase subunit TIM50	2.06
**O14880**	*MGST3*	Microsomal glutathione S-transferase 3	2.14
**P54108**	*CRIS3*	Cysteine-rich secretory protein 3	2.45
**O14657**	*TOR1B*	Torsin-1B	2.47
**Q08209**	*PP2BA*	Serine/threonine-protein phosphatase 2B catalytic subunit alpha isoform	2.58
**Q92905**	*CSN5*	COP9 signalosome complex subunit 5	2.98
**P09960**	*LKHA4*	Leukotriene A-4 hydrolase	3.14
**P21980**	*TGM2*	Protein-glutamine gamma-glutamyltransferase 2	3.19
**O95299**	*NDUAA*	NADH dehydrogenase [ubiquinone] 1 alpha subcomplex subunit 10	3.44
**P30626**	*SORCN*	Sorcin	3.60
**Q8TEK3**	*DOT1L*	Histone-lysine N-methyltransferase	3.62
**Q05639**	*EF1A2*	Metalloproteinase inhibitor 3	3.87
**Q9NZN4**	*EHD2*	Protein Nef	5.43
**O00148**	*DX39A*	ATP-dependent RNA helicase DDX39A	5.43
**Q9GZZ9**	*UBA5*	Ubiquitin-like modifier-activating enzyme 5	5.71
